# Letter to the editor: Methodological considerations regarding hospitalization rates for salmonellosis in Chile

**DOI:** 10.1016/j.pmedr.2026.103651

**Published:** 2026-09-25

**Authors:** Héctor Fuentes-Barría

**Affiliations:** Centro de Investigación en Medicina de Altura (CEIMA), Universidad Arturo Prat, Iquique, Chile.

Dear Editor,

We read with interest the study by Rojas Navia et al. (Rojas Navia et al., 2026), reporting nationwide hospitalization rates for salmonellosis in Chile between 2021 and 2024. The use of national administrative data provides valuable information on hospital-treated salmonellosis; however, several methodological issues warrant consideration when interpreting the reported estimates.

First, the reported overall hospitalization rate of 3.61 per 100,000 inhabitants requires clarification. The manuscript reports annual rates of 3.48, 3.25, 3.44, and 4.29 per 100,000 for 2021–2024, respectively (Rojas Navia et al., 2026). The overall value appears to represent an average of annual rates rather than a cumulative rate calculated using the total number of discharges and the corresponding population-time at risk. These approaches are not interchangeable, and the numerator-denominator framework used to derive the overall estimate should therefore be explicitly reported.

Second, greater detail regarding case ascertainment from the Department of Health Statistics and Information (DEIS) is needed. The manuscript does not specify the ICD-10 codes used, whether salmonellosis was required to be the principal diagnosis, or whether secondary diagnoses were included (Rojas Navia et al., 2026). This is important because administrative discharge diagnoses are imperfect proxies for laboratory-confirmed infection. Previous validation studies have demonstrated variable sensitivity and predictive value of ICD-coded hospital diagnoses, including substantial underascertainment of culture-confirmed *Salmonella* infections (Ma et al., 2008).

Third, the terminology of “hospitalization rate” and “patients” should be used cautiously when the underlying dataset consists of hospital discharge records. Unless individual-level identifiers permit exclusion of repeated admissions, the 2672 records reported by Rojas Navia et al. represent hospital discharges or episodes rather than necessarily 2672 unique patients Rojas Navia et al., 2026. This distinction is relevant when estimating population-level hospitalization burden.

Finally, the interpretation of a “post-pandemic increase” appears stronger than supported by the descriptive analysis. Only four annual observations were available, and no formal trend analysis was performed. Consequently, the increase observed in 2023–2024 should preferably be described as a temporal increase rather than attributed to reopening, mobility, healthcare normalization, or other pandemic-related mechanisms Rojas Navia et al., 2026. Time-series or interrupted time-series approaches would provide stronger evidence for such interpretations (Patel et al., 2023; Draper et al., 2026).

Clarifying these methodological aspects would improve the reproducibility and epidemiological interpretation of this important national analysis.

## CRediT authorship contribution statement

**Héctor Fuentes-Barría:** Conceptualization, Formal analysis, Investigation, Methodology, Resources, Visualization, Writing – original draft, Writing – review & editing.

## Informed consent

Not applicable, as no human participants were involved.

## Ethical approval

As this manuscript is a commentary, ethical approval was not required.

## Funding

The authors declare that no external funding was received for this work.

## Declaration of competing interest

The author declares that they have no known competing financial interests or personal relationships that could have appeared to influence the work reported in this paper.

## Data Availability

No new datasets were created or analyzed in this study.
